# Supplementary material for: Extrachromosomal circular DNA and structural variants highlight genome instability in Arabidopsis epigenetic mutants
Source: Nat Commun. 2023 Aug 28;14:5236. doi: 10.1038/s41467-023-41023-0 (PMC10462705; doi:10.1038/s41467-023-41023-0)
Supplement: Supplementary file 1 — Supplementary Information [file 41467_2023_41023_MOESM1_ESM.pdf]

Extrachromosomal circular DNA and structural variants highlight genome instability in *Arabidopsis* epigenetic mutants

Zhang et al.

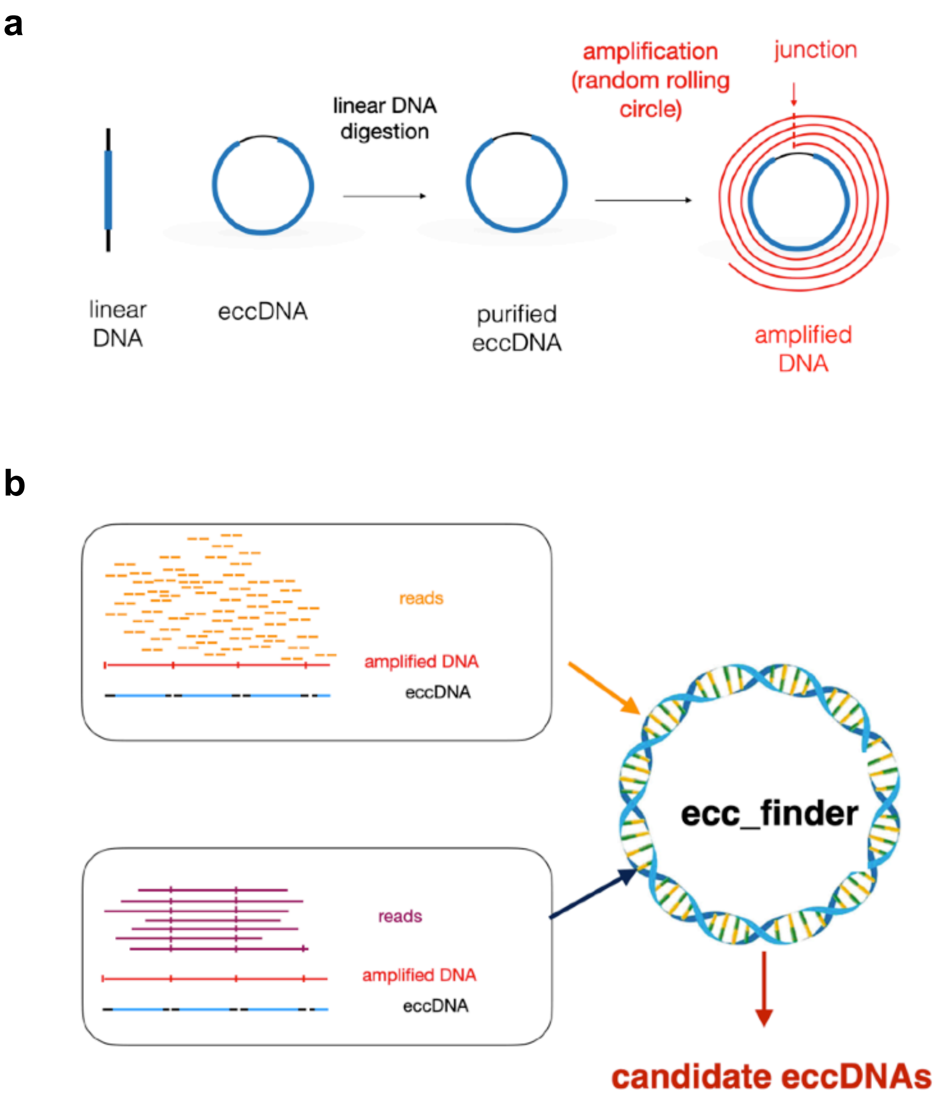

Supplementary Figure 1. Scheme illustrating the mobilome-seq approach used in this study.
(a) First DNA is extracted from plants and linear DNA (e.g. chromosomal DNA) digested using can exonuclease leading to an enrichment of eccDNAs. eccDNAs are then amplified using random rolling circle amplification with the Phi29 enzyme. **(b)** The Phi29 products are sequenced using short (Illumina Miseq) and long read (Nanopore MinION) sequencing platforms. ecc_finder together with homemade scripts are used to characterize the eccDNAs.

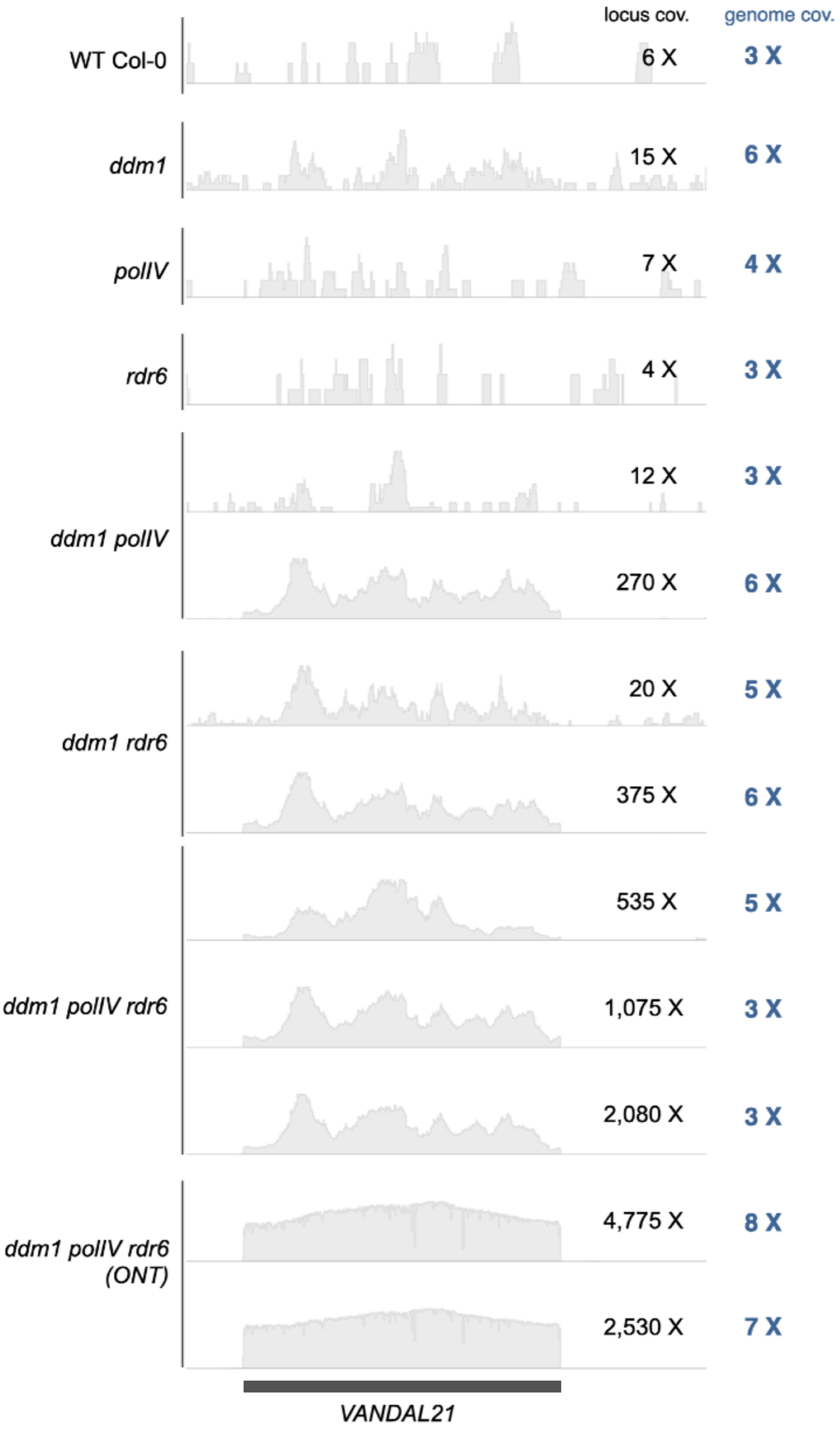

Supplementary Figure 2. Mobilome-seq data using Illumina and Nanopore sequencing (ONT) showing the coverage at a *VANDAL21* locus on chromosome 2. Mobilome-seq data in *ddm1*, *polIV, rdr6, ddm1 polIV*, *ddm1 rdr6* and *ddm1 rdr6 polIV* triple mutant plants using Illumina and Nanopore sequencing (ONT) showing the coverage at a *VANDAL21* locus (ID19197616, Chr2:10,002,636-10,008,008, *AT2TE42810*)

a

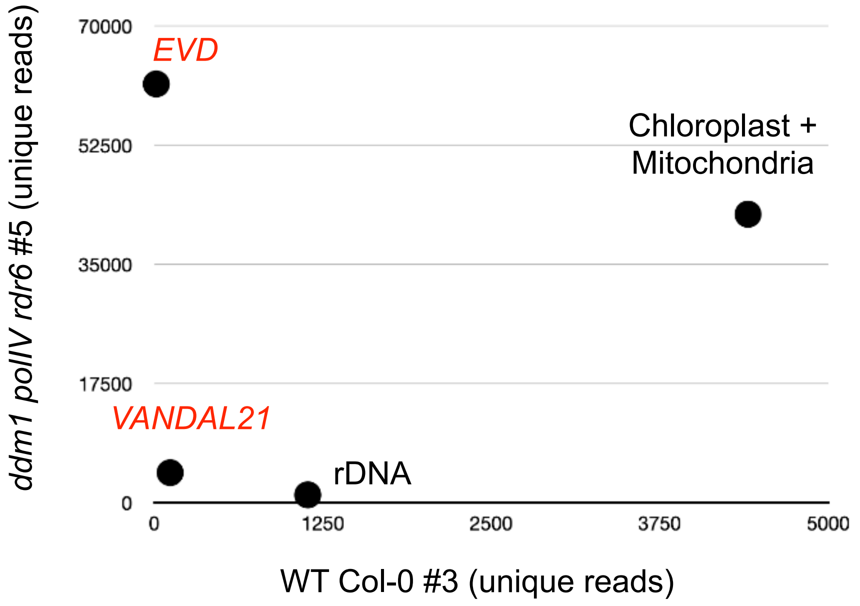

b

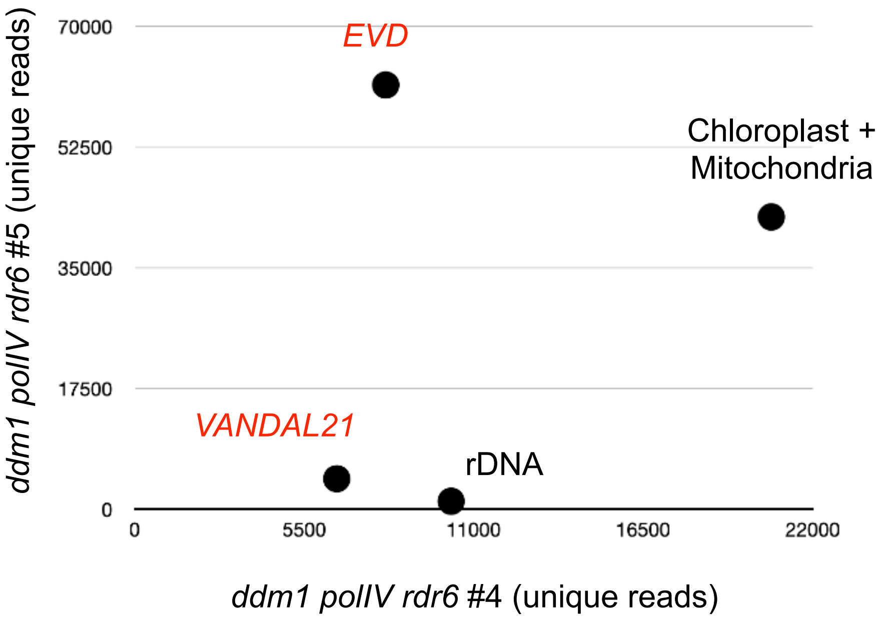

Supplementary Figure 3. ONT mobilome-seq data. Comparison of the number of unique reads mapping to rDNA (Chr2:1000-10559; Chr3:14191652-14205229), *EVD*, *VANDAL21* and chloroplast/mitochondria in the triple mutant versus the WT (**a**) and the two biological replicates of the triple mutants (**b**). Source data are provided as a Source Data file.

a

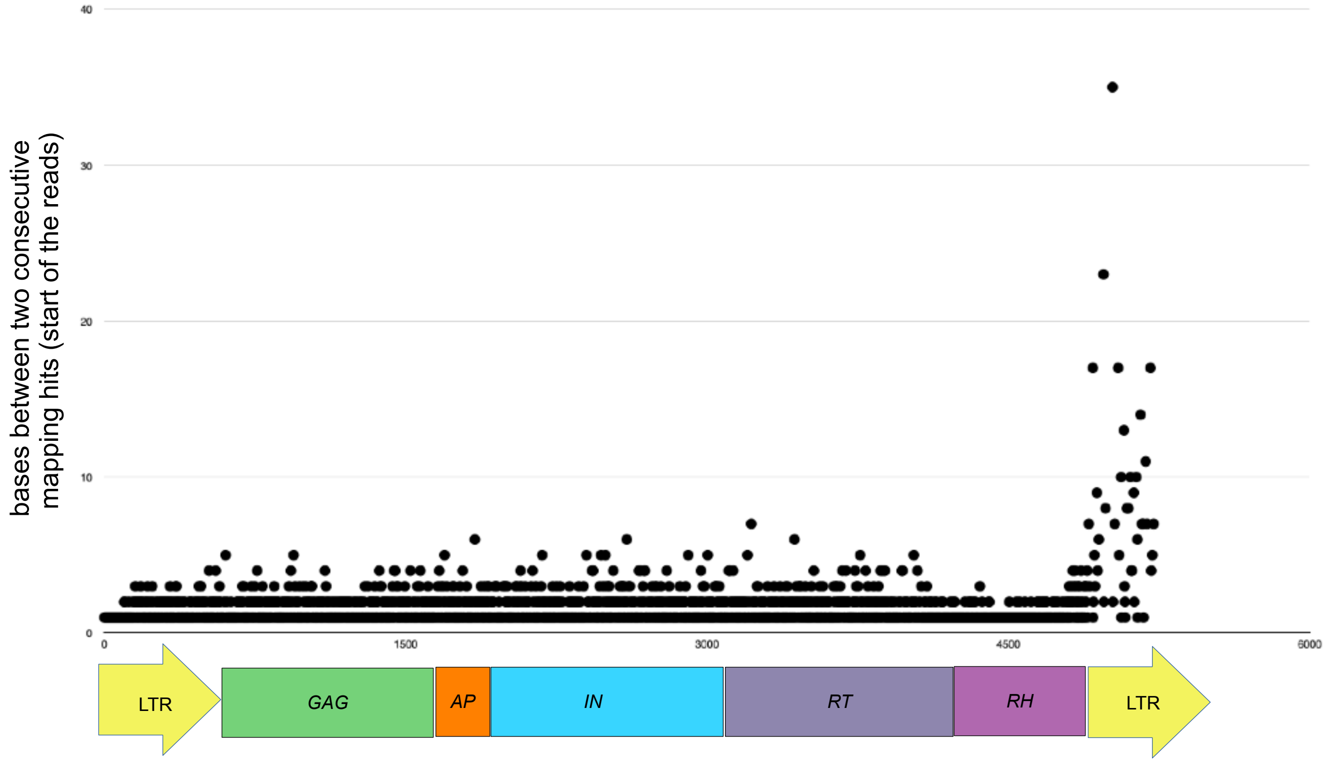

b

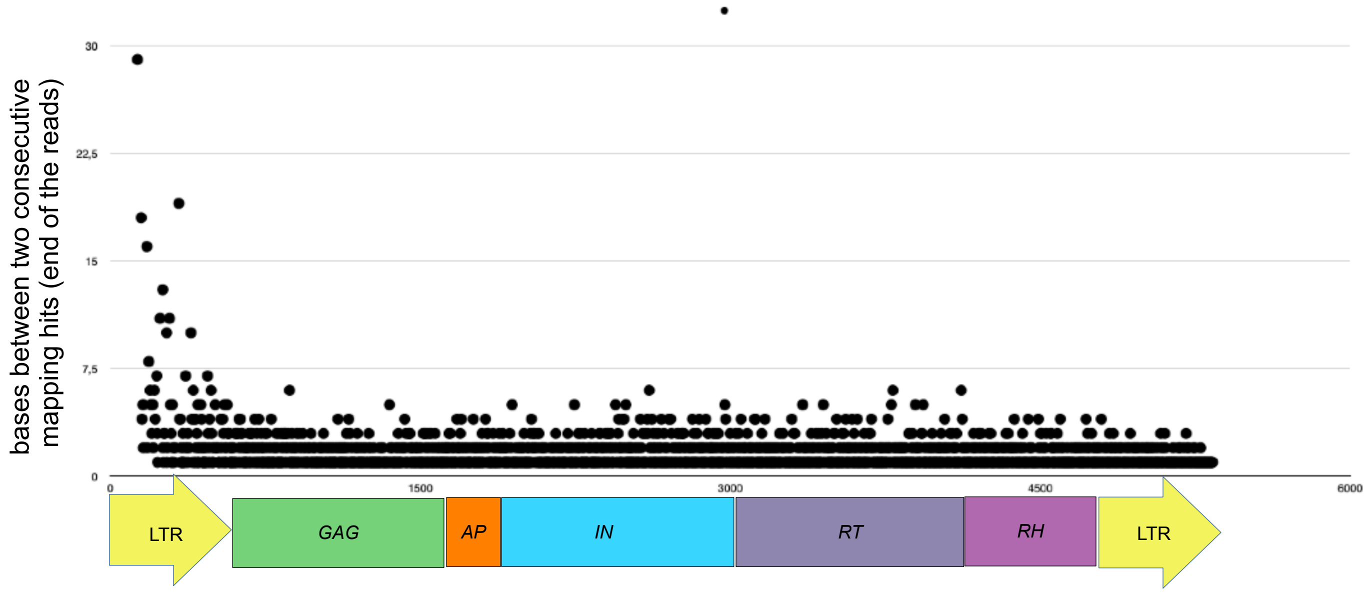

Supplementary Figure 4. Location of the start and end of ONT Mobilome-seq reads mapped on *EVD*. ONT mobilome reads from *ddm1 rdr6 polIV* were mapped on *EVD* sequence (*AT5G17125*) and filtered for unique reads and mapping quality above 50. The start and end position was scored for each read and the difference between consecutive mapping locations was plotted for start (**a**) and end (**b**) position. No hot spot for truncation could be detected. Note that mapping is more difficult at the LTRs because they are 100% identical.

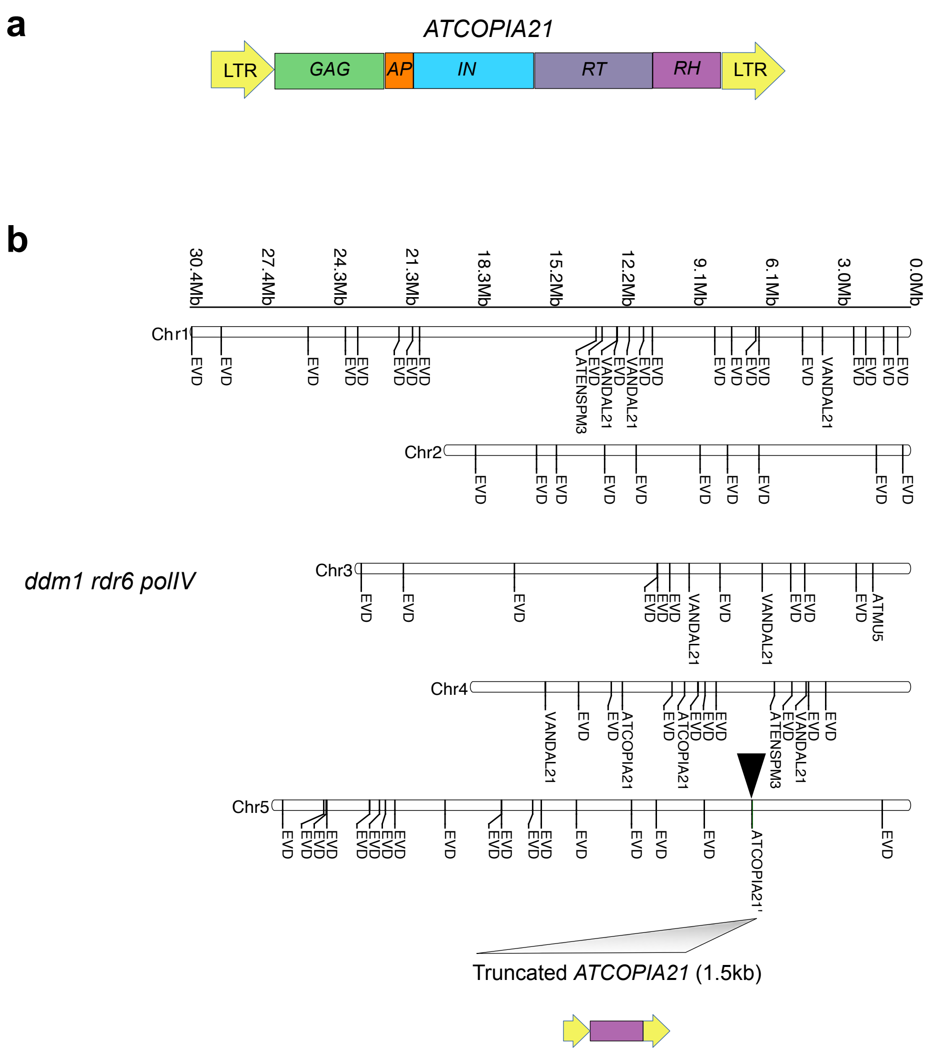

Supplementary Figure 5. A truncated *ATCOPIA21* new genomic copy is detected in *A. thaliana ddm1 polIV rdr6* mutant. (**a**) A schematic view of *ATCOPIA21* structure showing the main domains: LTR (yellow arrow), GAG (green), aspartic protease (AP, orange), integrate (IN, cyan), reverse transcriptase (RT, purple) and RNase H (RH, dark orchid). (**b**) Localisation of new TE genomic insertions in *ddm1 polIV rdr6* (insertions of *EVD*, *ATENSPM3*, *COPIA21*, *VANDAL21* and *ATMU5*). Truncated *ATCOPIA21* insertion on chromosome 5 is shown as a black triangle. Source data are provided as a Source Data file.

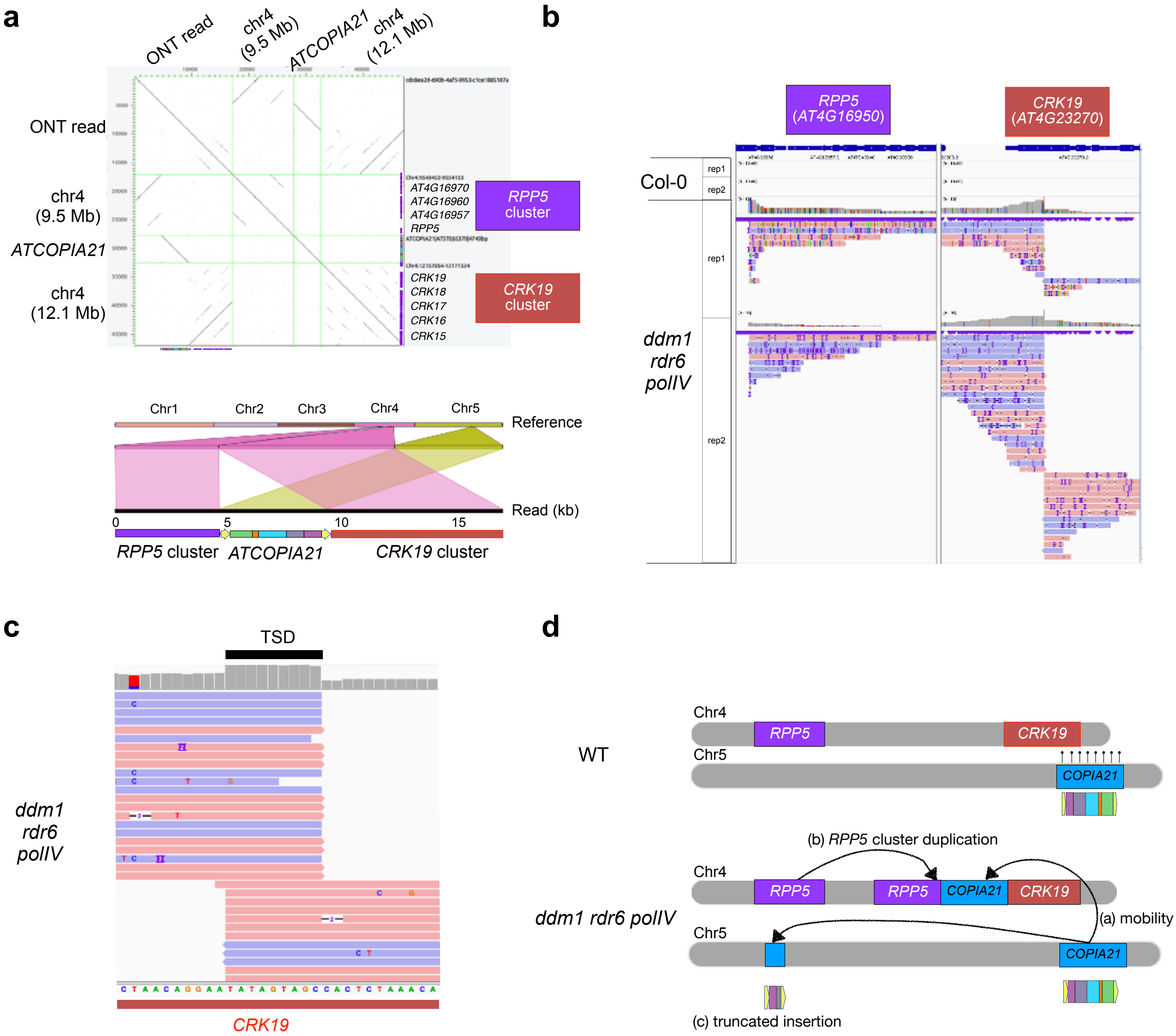

Supplementary Figure 6. *ATCOPIA21* mobility in the *A. thaliana ddm1 rdr6 polIV* triple mutant is associated with *RPP5* locus partial duplication. (**a**) Dot plot of an ONT read from *ddm1 rdr6 polIV* genome mapping to 3 different genomic loci as indicated. and graphical visualization of the data. The read shown is from run FAK64234 but similar chimeric reads were identified in a biological repeat (run FAK46256). (**b**) ONT genomic reads from *ddm1 rdr6 polIV* and containing *ATCOPIA21* were selected and mapped to the TAIR10 reference genome. These aligned reads are displayed on IGV at both the *RPP5* (*AT4G16950.* c. 9.5Mb on Chr4) and the *AT4G23270* loci (c. 12.1Mb on Chr4). (**c**) ONT reads showing a target site duplication (TSD) generated by *ATCOPIA21* insertion at the *CRK19* locus in *ddm1 rdr6 polIV* (15bp shown before and after 12,171,520 on chromosome 4), the TSD is visible thanks to read mapping locations ending or starting at the same position on the reference. (**d**) Possible scenario for the consequences of *ATCOPIA21* mobility (a) in *ddm1 rdr6 polIV* leading to (b) *RPP5* locus duplication and (c) truncated new insertions.

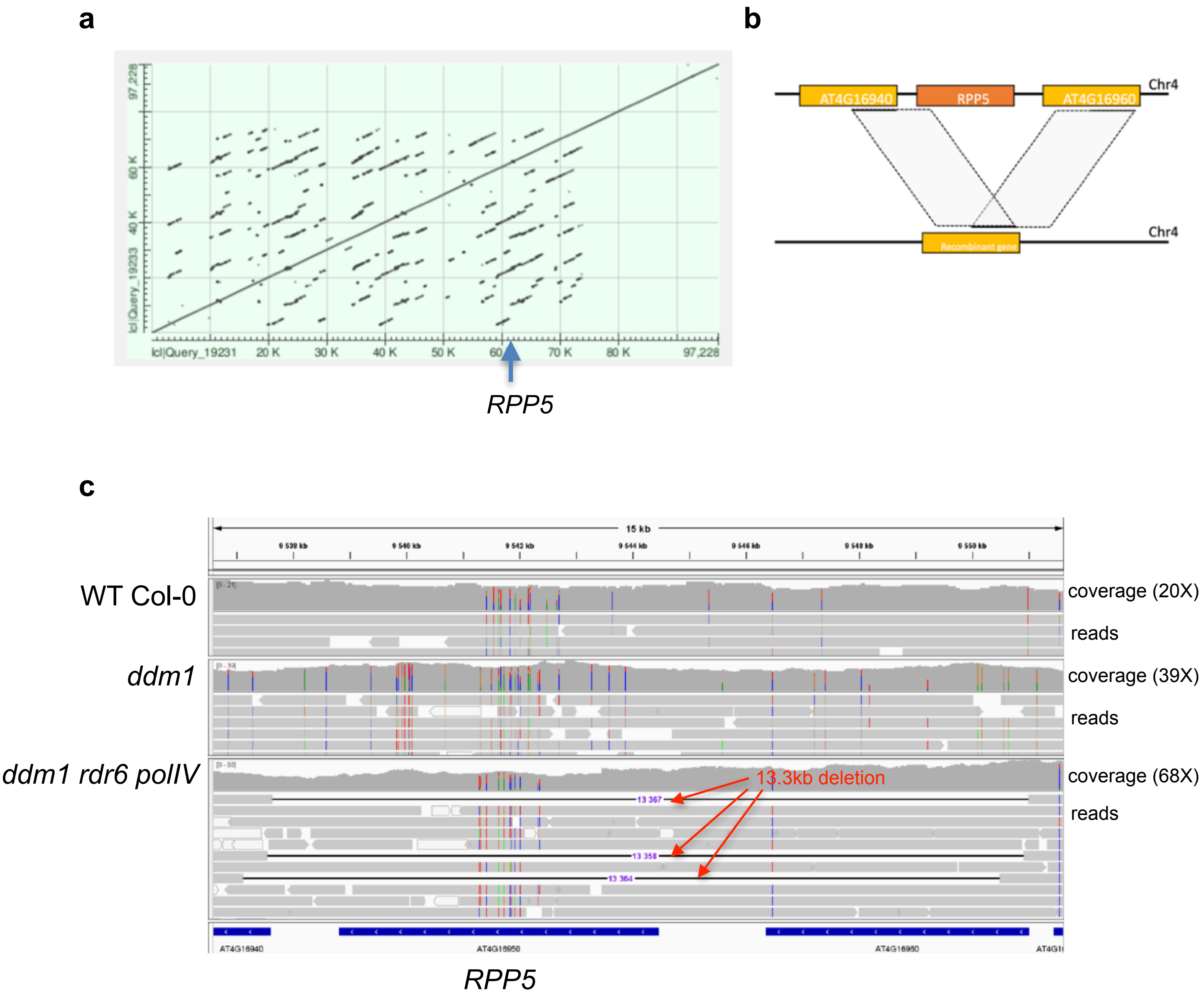

Supplementary Figure 7. Structural variation in *ddm1 rdr6 polIV* mutant at the *RPP5* locus. (**a**) Dotplot of the Col-0 reference genomic region surrounding the *RPP5* NLR cluster (10kb upstream *At4g16860/RPP4* to 10kb downstream *At4g16990/RLM3)*. (**b**) Scheme indicating how the *RPP5* locus recombined. (**c**) IGV view showing the reads coverage for three samples: WT, *ddm1* and the triple mutant *ddm1 rdr6 polIV* (replicate#2, FAK64234) at the *RPP5* locus (*AT4G1950*). Note the 3 Nanopore reads indicating a 13.3 kb deletion in the triple mutant. The sequencing was performed from a pool of 5-6 plants for this sample.

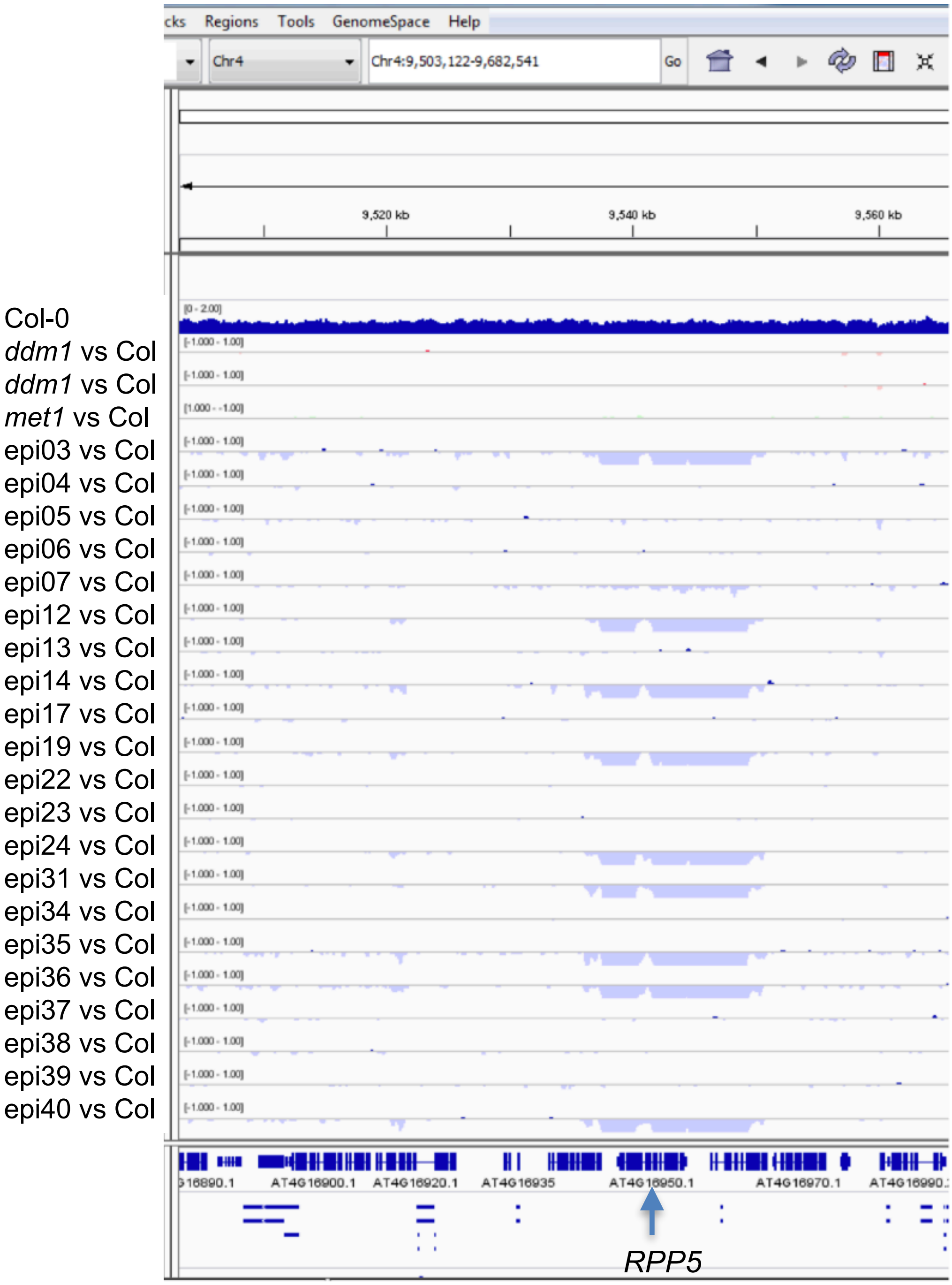

Supplementary Figure 8. Genomic instability at the *RPP5* locus in the *met1*-derived epiRIL population. IGV view of the *RPP5* locus in epiRILs. Reads corresponding to each genotype were mapped to the reference genome and compared to the WT control. Apart from Col-0, only fold change leaves compared to WT are shown. A light blue signal below the line indicates a lack of coverage of the corresponding locus. Of note, the sequencing libraries were PCR free, allowing for a better quantification (see Catoni et al. 2019).

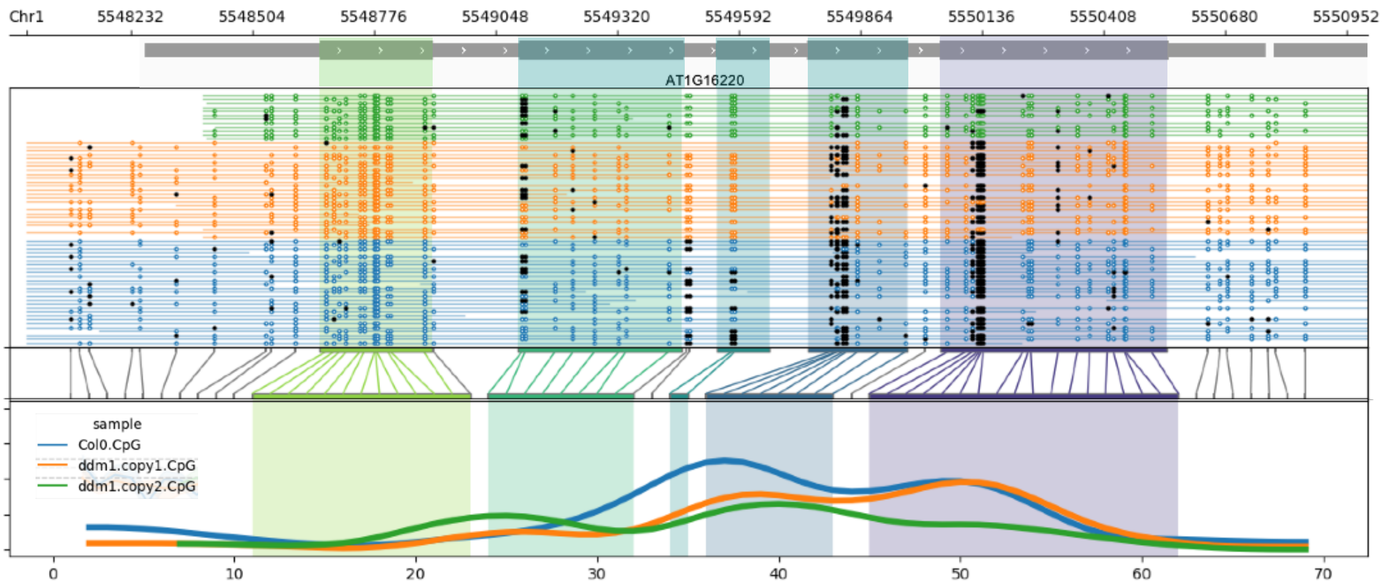

Supplementary Figure 9. Methylation levels at the genomic 55kb tandem duplication on chromosome 1 in a *ddm1* mutant plant. Cytosine methylation at the duplicated gene at the start of the 55kb duplication on Chromosome 1. From top to bottom: the plot shows genome coordinates, gene transcripts, ONT read mapping with modified bases as closed (methylated) or open (unmethylated) circles and smoothed methylated fraction plot. Exons of the *AT1G16220* gene are highlighted. DNA methylation data are given for Col-0 (blue), first tandem repeat in *ddm1* (orange) and second tandem repeat in *ddm1* (green).

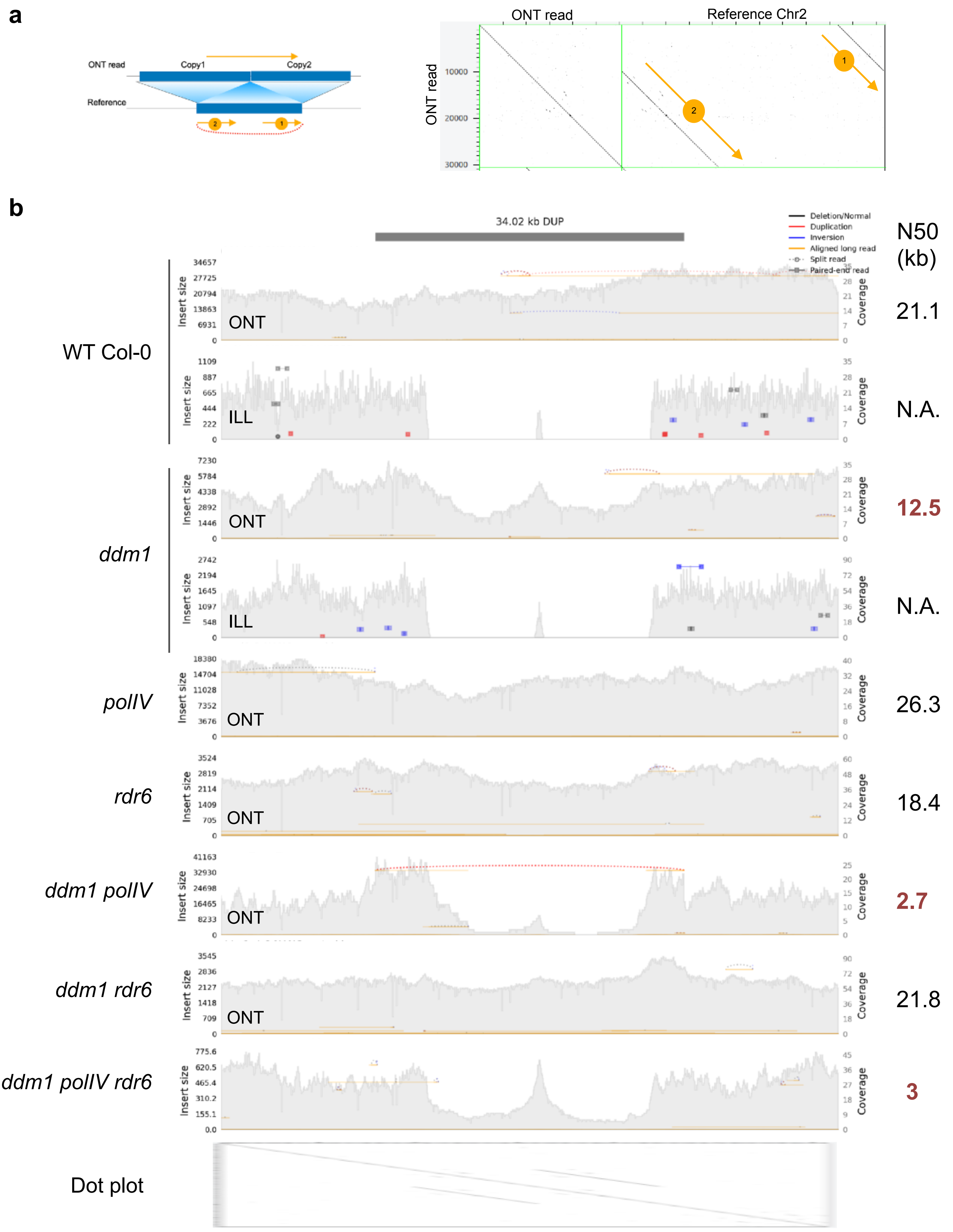

Supplementary Figure 10. Detection of a 34kb genomic duplication in *ddm1 polIV* mutant plants. (**a**) Scheme indicating how the ONT reads (orange arrows) spanning the junction of two tandem repeats will be split and aligned on the reference and Dot plot showing a raw 30kb long ONT read (808afc59-1a97-403a-b7c5-cedce28d8a55) versus the reference at the duplicated region on Chr2 (Chr2: 231518-287731). (**b**) Examples of read depth for genomic reads aligned to the TAIR10 reference genome on chromosome 2 at the duplicated locus shown in A, for *A. thaliana* wild type Col-0 and mutant genotypes as indicated on the left. Dotted red lines and grey rectangle highlight the duplication as detected by Sniffles. A dot plot is shown at the bottom to illustrate the mapping problem encountered due to a tandem duplication already existing in the reference genome. Note that ONT data with low N50 (indicated in red) do not allow to span the duplication in the reference genome.
